# Misspecifications of Stimulus Presentation Durations in Experimental Psychology: A Systematic Review of the Psychophysics Literature

**DOI:** 10.1371/journal.pone.0012792

**Published:** 2010-09-29

**Authors:** Tobias Elze

**Affiliations:** Research Group Complex Structures in Biology and Cognition, Max Planck Institute for Mathematics in the Sciences, Leipzig, Germany; The University of Western Ontario, Canada

## Abstract

**Background:**

In visual psychophysics, precise display timing, particularly for brief stimulus presentations, is often required. The aim of this study was to systematically review the commonly applied methods for the computation of stimulus durations in psychophysical experiments and to contrast them with the true luminance signals of stimuli on computer displays.

**Methodology/Principal Findings:**

In a first step, we systematically scanned the citation index Web of Science for studies with experiments with stimulus presentations for brief durations. Articles which appeared between 2003 and 2009 in three different journals were taken into account if they contained experiments with stimuli presented for less than 50 milliseconds. The 79 articles that matched these criteria were reviewed for their method of calculating stimulus durations. For those 75 studies where the method was either given or could be inferred, stimulus durations were calculated by the sum of frames (SOF) method. In a second step, we describe the luminance signal properties of the two monitor technologies which were used in the reviewed studies, namely cathode ray tube (CRT) and liquid crystal display (LCD) monitors. We show that SOF is inappropriate for brief stimulus presentations on both of these technologies. In extreme cases, SOF specifications and true stimulus durations are even unrelated. Furthermore, the luminance signals of the two monitor technologies are so fundamentally different that the duration of briefly presented stimuli cannot be calculated by a single method for both technologies. Statistics over stimulus durations given in the reviewed studies are discussed with respect to different duration calculation methods.

**Conclusions/Significance:**

The SOF method for duration specification which was clearly dominating in the reviewed studies leads to serious misspecifications particularly for brief stimulus presentations. We strongly discourage its use for brief stimulus presentations on CRT and LCD monitors.

## Introduction

### Motivation and Scope

Precise timing of visual stimuli can be a requirement for experiments in psychology. For instance, in rapid serial visual presentation paradigms, the onsets and offsets of the single stimuli need to be known and controlled for exactly to study perceptual phenomena like repetition blindness [1] or the attentional blink [2]. Another widely used technique is visual masking [3] in which the visibility of a briefly presented target stimulus is impaired by a masking stimulus presented in close spatiotemporal proximity. This paradigm, which is used in many experimental situations in psychophysics and visual neuroscience, requires brief stimulus presenations and precise timing not only for the stimuli themselves but also for the temporal distances between their presentations.

Many experimenters from these fields work with common computer hardware to display their stimuli and specify the presentation times according to common beliefs about how their display devices work. The present study shows that these widely applied methods of calculating presentation times bear severe misconceptions. Some of the specified times are even unrelated to the actual presentation durations.

This work is composed of two parts. First, a systematic review of experimental articles with brief presentation of visual stimuli investigates what temporal measures are commonly used for presentation durations. Second, these duration specification methods and their underlying assumptions are contrasted with the true temporal signals of the two most frequently used types of computer monitors, namely cathode ray tube (CRT) monitors and liquid crystal displays (LCD).

In the following paragraphs a concise introduction to common computer monitors and standard ways how to control them is given, including a brief review of the key literature.

### Operating standard computer monitors

The graphics adapter of the computer addresses the visual image on the monitor as a discrete raster of pixels. As CRT monitors have been the dominating display technology over decades, many of the concepts how to control a monitor are taylored to the CRT technology. The main characteristics of this technology have been described already decades ago [4], [5]: An electron beam inside the cathode ray tube scans the raster of pixels linewise from left to right, beginning with the uppermost line. When the beam has traversed the rightmost pixel in a line above the last line then it jumps to the first pixel one line below.

**Figure 1:**
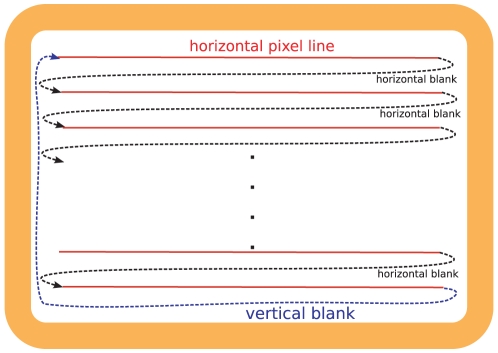
Schematic of the CRT raster scan. The dashed arrows depict the course of the electron beam. The duration of the vertical blank is about 5% of the frame.

When the beam has passed the rightmost pixel in the last line it jumps back to the leftmost pixel in the first line. The period of time of this jump we call *vertical blank*. Fig. 1 outlines the raster scan concept. In the following, we call the time between two vertical blanks a *frame*. The duration of the frame is the reciprocal of the *refresh rate* of the monitor. The frequently used 60 Hz refresh rate, for instance, results in frames of 16.7 ms.

These CRT characteristics determine how CRT monitors can be controlled: The graphics adapter of the computer needs to be synchronized to the vertical blank of the monitor during which the buffered signal of the graphics card is sent. Therefore, the frame is the essential temporal unit to control stimulus durations.

LCD monitors do not have electron beams and the LCD technology would theoretically allow several other modes of display refresh. However, for historical reasons, LCD panels conform to the same raster scan mechanism as CRTs.

### Temporal signals on CRT and LCD devices

**Figure 2:**
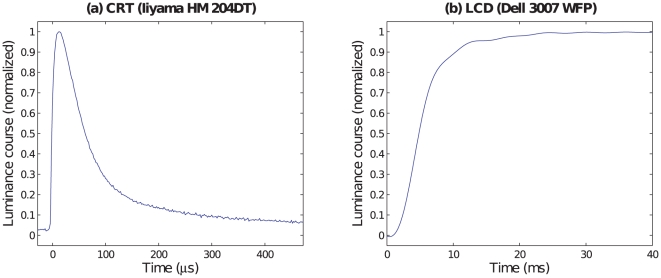
Comparison of CRT and LCD luminance transition signals. The plots show the onset of a green stimulus on a black monitor at time 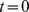. The CRT signal shown in (**a**) was measured from an Iiyama HM 204 DT monitor. The LCD signal shown in (**b**) was taken from a Dell 3007 WFP panel after backlight filtering by the division method [13]. Note the different scalings of the abscissas.

Temporal signals of CRT monitors have been extensively studied in vision science [4]–[11]. The single dots of a CRT are covered with a substance called *phosphor*. Upon stimulation, the luminance of the phosphor rises rapidly and reaches its maximum almost instantaneously. After this, the energy decays, initially exponentially, later on converging to a power law course. There are different phosphors. On modern consumer CRT monitors, the most frequently used phosphor is P22. Fig. 2(a) shows the recording of the luminance course of a P22 phosphor after stimulation at time 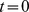.

The time course of the phosphor decay is an essential characteristic of a CRT monitor. Because of the non–exponential course, specifications of decay constants, although convenient, are not appropriate for decays to low percentages of the maximum. Usually, decay times to the 10% level are specified. The persistence of the luminance signal depends on the phosphor type. For the frequently used P22 phosphor, Sherr [6] (p. 91) specifies decay times ranging from 1.5 ms to 6 ms. In our illustrative measurement (Fig. 2(a)), the luminance falls below the 10% level already after about 400 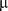
s. Note that a stimulus presented for 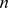 frames is not displayed as a constant signal but rather as a succession of 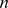 such pulses as shown in Fig. 2(a).

Compared to the CRT literature, up to now there is substantially less literature about temporal signals of LCD monitors, and the use of LCD devices for medical and vision science purposes has been studied only by a few recent works [11]–[13]. In contrast to CRT signals, LCD signals are not pulsed but sample and hold displays, that is, the signal can be “switched on”, stays on a constant level, and can be “switched off” again. LCD panels do not have phosphors but are passively lit by a steady backlight. This backlight is not a constant signal but subject to periodic modulations the effects of which have been recently discussed [13], [14]. LCD panels control their luminance by aligning the optical axis of the liquid crystal to allow transmission of the backlight through polarizers. Luminance changes on LCD monitors are determined by the speed of the liquid crystal alignment. The duration of an LCD luminance transition is called *response time*.

Fig. 2(b) shows the recording of a luminance transition from black to green on an LCD panel. The backlight modulations have been filtered by the division method [13].

**Figure 3:**
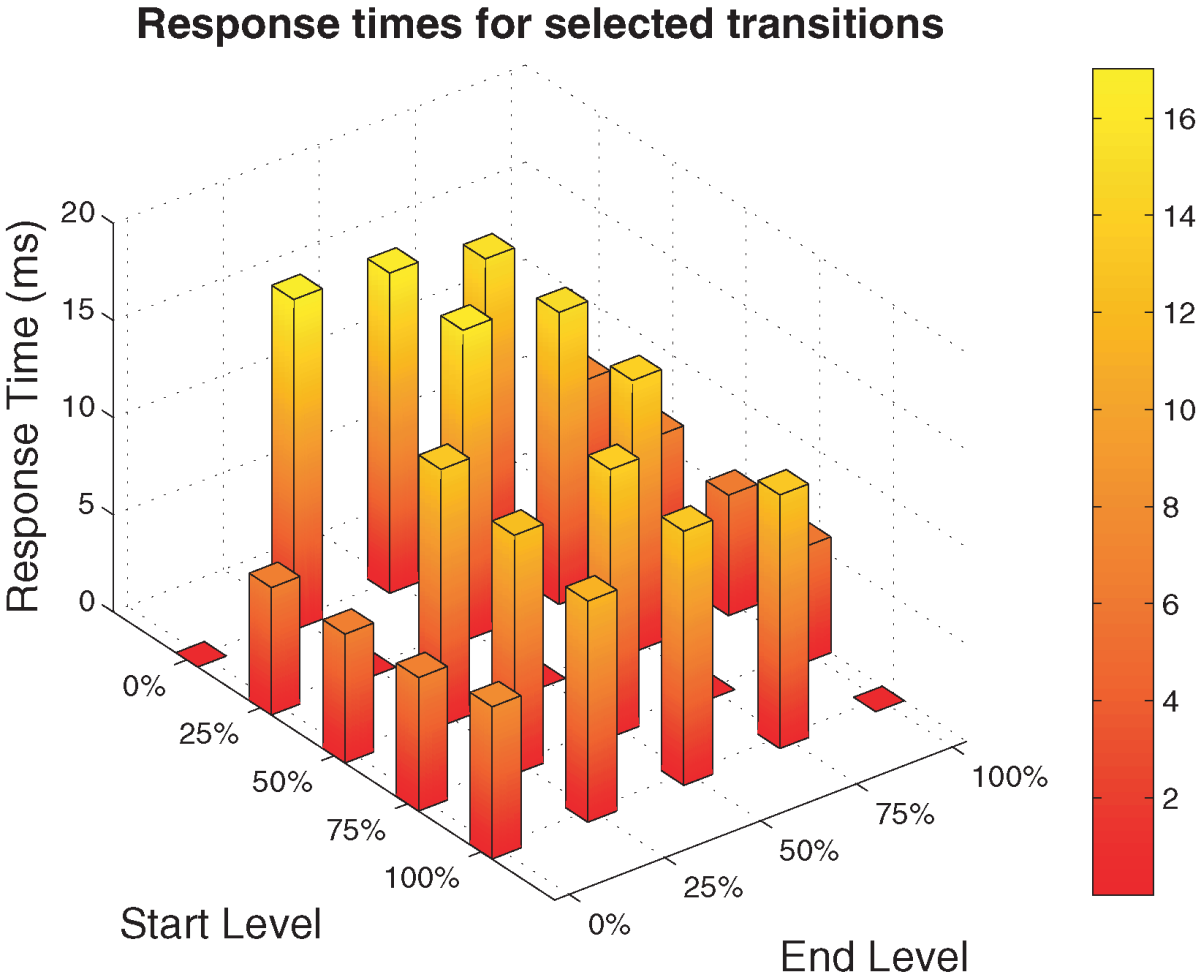
Response times variability over different luminance levels (Dell 3007 WFP LCD panel, green channel). The bar plots show averaged response times over five measurements. Response times have been measured from five different initial levels (given as percentage of the maximal luminance of the monitor) to five target levels. All response times have been calculated by means of the division method with dynamical filtering [13].

Note that the response times vary not only over different monitor models but are not even homogeneous with respect to different luminance levels on a single monitor [11]–[13]. Fig. 3 shows an example for this variability. The response times of a Dell 3007 WFP monitor have been measured for luminance transitions between 0% and 100% of the maximal luminance of the monitor in five steps. For this monitor, response times tend to be smallest for transitions to the target level 0 (black) and to the target level 100% (white). Note that transitions between two distinct levels need not be symmetric. For instance, the response time of the 25%
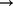
0% transition is only a fraction of the response time of the 0%
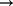
25% transition.

## Results

**Figure 4:**
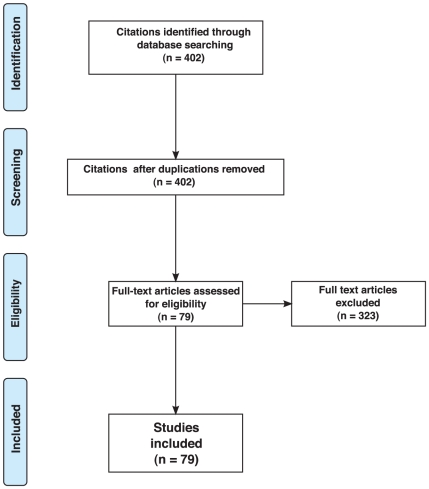
Flowchart for study selection.

The search of the electronic data base according to the criteria given in the Methods section produced a total of 402 citations. All citations were unique. Of these citations, 323 studies were discarded because after reviewing the full text it appeared that the experiments described in them did not make use of stimulus durations, onset asynchronies, or interstimulus intervals of less than 50 ms. Fig. 4 shows the respective flowchart according to the PRISMA guidelines [15], [16].

The remaining 79 studies, which included a wide range of topics (e.g. attentional blink, metacontrast, apparent motion, flash lag effect, letter or word identification, temporal order judgment, face recognition, change and repetition blindness, or macula degeneration research), matched all the given criteria. Of these, only 17 (21.5%) described how they calculated their stimulus presentation durations. All of them summed up the frames during which the stimulus was presented. In the following we call this measurement *sum of frames* (SOF) method. For 58 further studies the SOF measure could be deduced from their given presentation duration values, either because their monitor refresh rate was specified or because all values were integer multiples of typical intervals between two monitor refreshes. For the remaining works no measurement method could be inferred from their given data.

36 (45.6%) articles mentioned the monitor model used. Two of them used an LCD panel, all the others used CRT monitors. 25 (31.6%) of the studies used stimulus duration or the interval between two stimuli as critical variables. Two studies verified their duration specifications by additional photodiode/oscilloscope measurements: One of them [17] verified the temporal interval between stimulus onsets and the other one [18] checked the monitor's refresh rate.

5 works computed statistics over presentation durations. Three of them [19]–[21] calculated means of durations, the other two works [22], [23] fitted psychometric functions.

**Figure 5:**
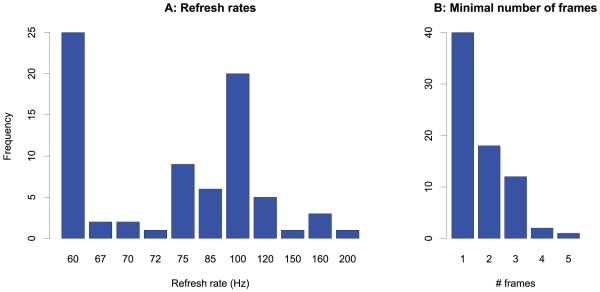
Display timing characteristics in the literature review studies. Frequency of refresh rates (A) and the minimal numbers of frames (B) used in the experiments.

Fig. 5 shows the frequencies of the refresh rates (A) and of the minimal numbers of frames used for the shortest duration specification for all the reviewed studies where these parameters were either specified or could be inferred from the given stimulus durations. The figure illustrates that more than half of the studies (50.6%) worked with minimal presentation times as short as one single frame. Furthermore, the most frequently used refresh rate is as low as 60 Hz which allows a temporal resolution of only 16.7 ms.

## Discussion

### Possible selection bias

The restriction to three journals and two search terms might be criticized with respect to a possible selection bias of the reviewed studies. However, the selected journals are key journals in the fields of experimental psychology (*Journal of Experimental Psychology: Human Perception and Performance* and *Journal of Experimental Psychology: Learning, Memory, and Cognition*) and vision science (*Vision Research*).

In addition, the choice of two frequently used experimental techniques as search terms instead of searching for special perceptual phenomena/illusions aims to minimize the selection bias with respect to research topics. Indeed, the selected studies contain a wide range of psychological subjects, as noted in the Results section.

### The SOF method and its misconceptions

All stimulus duration specifications for which a specification method could be found out were given according to the SOF measure. This finding is surprising for the at least 34 studies which used CRT monitors since for the latter devices the SOF method has been criticized already more than a decade ago [9]. The signal plot in Fig. 2(a) illustrates why this method is not suitable for CRT monitors: The short pulse of the CRT luminance signal occurs at the very beginning of the frame. After this pulse, the luminance is nearly zero up to the start of the subsequent frame. The SOF method, however, implicitly assumes a rectangular signal which starts at the beginning of the onset frame of the stimulus and lasts until the end of the offset frame.

**Figure 6:**
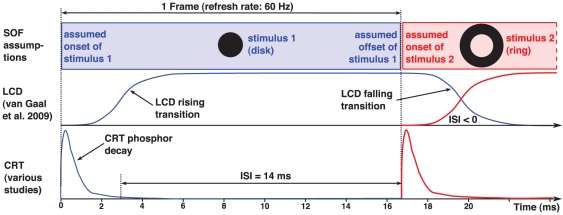
Stimulus succession on CRT vs. LCD monitors. Schematic of the luminance course of a disk stimulus presented for one frame followed by a surrounding ring stimulus. The signals of the disk stimulus are shown in blue, those of the ring stimulus in red. The top row outlines the signal assumptions according to the SOF method. The two underneath rows sketch the true signal shapes on LCD and for CRT monitors, respectively.

Fig. 6 compares the SOF assumptions with true CRT and LCD luminance signals and the related implications for a hypothetical psychological experiment with brief stimulus presentation. We assume the classical disk–ring paradigm which was introduced already decades ago [24]. Variations of this paradigm were used in several of the studies from the literature review, both for CRT monitors [25] and for LCD monitors [26].

In our hypothetical experiment, a disk stimulus is presented for one frame. In the next frame, a surrounding ring is presented, and our hypothetical experimenter specifies both the stimulus duration and the interstimulus interval (ISI) between disk and ring according to the SOF method. As for the ISI, the SOF method assumes temporal adjacency, that is the onset of the ring occurs at the same time as the offset of the disk. The experimenter would therefore state an ISI of zero and a duration of the disk of one frame, as outlined in the top row of Fig. 6. If we assume the refresh rate which was most frequently used in the reviewed studies, namely 60 Hz (see Fig. 5A), the experimenter would specify a stimulus duration of 16.7 ms.

Let us compare these assumptions with the signal shapes on a CRT monitor, as sketched in the bottom row of Fig. 6. The luminance signal of the disk stimulus is a pulse determined by the almost instantaneous phosphor activation at frame start and by the following phosphor decay to zero. Obviously, it is unclear how to specify the true stimulus duration. One might integrate over the pulse and approximate a rectangular signal of the equivalent luminance or one might define the offset at the point in time when the luminance has decayed to 10% of its maximum, or define a time 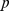 from the beginning of the frame until the luminance has decayed to nearly zero, as Bridgeman [9] suggested. In Fig. 6 we do the latter. In our sketch, 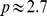 ms, which is in the time range of typical CRT phosphors [6]. The true disk duration would be 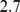 ms now instead of 16.7 ms, that is, the SOF method would considerably overestimate the duration. Note that there are monitors with much lower phosphor decay times (see, for instance, Fig. 2(a)). In addition, the assumption of temporal adjacency is violated, as the true ISI is 14 ms and hence substantially greater than zero.

The LCD signal as sketched in the figure seems to be closer to the SOF assumption. However, this is only true if two conditions are met. First, the response times must be considerably shorter than one frame. This condition is often violated. It can take several frames until the signal reaches its target level [11]. Second, the rising and falling response signals must be symmetric. This need not be the case as well, as discussed above and illustrated in Fig. 3. In this case, the SOF measure might be improved by subtracting the rise time and adding the fall time if the first condition is met. The figure outlines as well that the ISI  =  0 assumption of the SOF measure is violated for LCD panels because the rising signal of the ring and the falling signal of the disk partially overlap, and there might be unexpected display effects particularly if the rising and the falling signals are not symmetric.

In addition, Fig. 3 illustrates that CRT and LCD signals are so different that it is not appropriate to use one and the same method to specify durations of brief stimuli.

Finally, it has to be noted that for CRT monitors the SOF method is suitable if distances between stimuli are not given in terms of ISI but in terms of stimulus onset asynchronies (SOA) instead. This holds for LCD monitors as well as long as the rising transition signals of the two stimuli are similar enough.

### Statistics over stimulus durations

For most of the literature review studies the wrong assumptions about stimulus durations probably do not have any impact on the experimental findings and conclusions. However, in studies where statistics are calculated over stimulus durations or ISIs, alternative timing specification methods may easily affect the experimental results after the statistical data analysis.

As mentioned above, such statistical analyses have been performed by five studies [19]–[23]. In the following, let us compare the durations 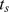 specified by the authors with respective durations 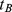 calculated according to the more accurate Bridgeman model which is described in detail in the next section.

Three works [19]–[21] calculated basic statistics like means and corresponding standard deviations. As 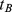 is almost one frame less than 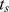, 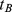 means are smaller than 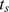 means, and for brief durations the difference is substantial. Moreover, 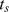 times are always multiples of one frame, but the Bridgeman model destroys this multiplicity. This can become relevant for stimulus duration comparisons, as the following example demonstrates.

Lleras and Moore [19] compared average presentation durations for a certain performance level under two conditions. Consistent with their hypothesis, in one of the conditions presentation duration was longer. They state the durations for the two conditions with 20.8 ms resp. 28.3 ms and report an increase of 39%. Their frame rate was 60 Hz, and their calculations of the averages were the results of different distributions of presentations for one and two frames (SOF measure: 16.7 ms resp. 33.4 ms) over 60 presentations in each condition. If we apply 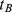, the 16.7 ms turn into phosphor decay 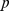 (only a few milliseconds) and the 33.4 ms into 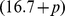 ms. Because of the violated proportionality, the increase will be much higher than their stated 39%.

In this case, applying the Bridgeman model gives even more evidence for their hypothesis, since an increase was predicted, but the “true” increase was even stronger than the assumed one. However, in cases where an increase according to the SOF measure would be so small that it is regarded to be irrelevant, the Bridgeman model could easily rise the difference above a possible significance level.

The fit of psychometric functions to stimulus durations, as practiced by some of the reviewed studies, requires careful considerations regarding the duration specification method as well, particularly in cases of parametric models which do not include vertical shifts of the data. Note that is has recently been shown for logistic functions that the loglikelihood difference of two conditions can even change its sign if durations are calculated according to the Bridgeman model [11]. That means, experimenters might assume opposite experimental results after their data analyses.

To sum up, different duration specification models can have considerable influences on statistical data analyses.

### Alternatives to the SOF method

For CRT monitors, alternatives to the SOF method have been discussed in the literature. Robson [8] suggests directed luminance changes to realize “perceptual stimulus durations” which differ from physical durations calculated according to SOF. For instance, the author reports that halving the luminance in the last frame of a stimulus that extends over 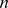 of frames can result in a retinal response the decay of which occurs halfway between the decay of the signal of 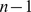 and 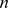 full luminance screens. This way, the experimenter can generate “perceptual” durations which are not multiples of frames.

While this is an interesting approach which may overcome the discretization limitations of stimulus durations, it requires reliable models of the visual system and goes far beyond the target of many experimenters who want to describe the physical durations of their stimuli.

Bridgeman [9] favors this type of description of the physical duration and suggests an approach which we will call *Bridgeman model* in the following. The Bridgeman model calculates the stimulus duration on a CRT monitor by SOF minus one frame plus the phosphor decay time. For stimulus presentations over many frames, the Bridgeman model converges to SOF. For brief durations, however, there are substantial deviations. Note that for single frame presentations *the stimulus duration according to the Bridgeman model is even unrelated to the duration according to SOF*, and the majority of the studies from the literature review used minimal presentation durations of one frame (see Fig. 5(b)).

While the Bridgeman model considers the specific luminance signal shape of CRT monitors, it neglects possible effects of the duty cycle (that is the ratio of the ON–period to the desired stimulus duration). For instance, for a two–frame CRT stimulus with a refresh rate of 60 Hz and a phosphor decay of 3.3 ms it calculates a stimulus duration of 20 ms although the ON–period of the signal was only 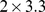 ms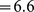 ms. Apart from that, it does not consider the special signal shape. For single frame presentations, the signal is a decaying pulse. The Bridgeman model defines the stimulus duration by the period during which the signal is different from zero and neglects the substantial luminance changes during this period.

In addition, as Bridgeman points out himself [9], the Bridgeman model neglects phase delays between the top and the bottom of the display. These delays are relevant particularly for single frame presentations. As outlined in Fig. 1, it takes almost one frame to build up the display from the topmost line to the last line. A stimulus presented at the bottom of the screen in frame 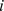 is temporally close to a stimulus presented at the top of the screen in frame 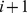.

For brief stimulus presentations it might be more useful to avoid the specification of durations in milliseconds. Instead, experimenters should report the durations by the number of frames only and give as many details as possible about the display device and its characteristics, like phosphor decay for CRTs or response times for LCDs.

### Conclusion

This systematic review shows that the SOF method is the clearly dominating measure to specify the durations of stimuli even for brief presentations. Furthermore, it is demonstrated why this method is inadequate for the two major display technologies, namely CRT and LCD, and that the luminance signals of these two technologies are fundamentally different. The use of the SOF method is strongly discouraged for brief stimulus presentations. Instead, experimenters should always specify the number of presentation frames, the monitor model and technology, the refresh rate, and ideally also details about the luminance signal shape (phosphor decay/liquid crystal response).

## Methods

### Literature review

The citation index Web of Science (Thomson Reuters) was systematically scanned for works with brief presentations of visual stimuli. For literature reviews, the choice of an appropriate search term is essential. The search term “brief presentation” did not prove useful, and searching for special perceptual phenomena like “attentional blink” might result in biased samples of works. Therefore, we decided our search terms to be two widespread techniques which are used to investigate a wide range of perceptual phenomena and which frequently require brief stimulus presentations. Namely, we chose articles which contained at least one of the two terms “rapid serial visual presentation” or “masking” either in title, abstract, or keywords.

All articles which met the search term criteria and appeared between 2003 and 2009 in one of the three journals *Vision Research*, *Journal of Experimental Psychology: Human Perception and Performance*, and *Journal of Experimental Psychology: Learning, Memory, and Cognition* were chosen if they contained experiments with stimuli presented for less than 50 ms or temporal distances between stimuli (SOA or ISI) of less than 50 ms on computer controlled displays. The latter criterion was checked for by reviewing the full text of all the studies obtained by the data base search.

This review was performed according to the Preferred Reporting Items for Systematic Reviews and Meta–Analyses (PRISMA) guidelines [15], [16].

### Signal measurements

In order to illustrate our analysis of signal shapes, we provide examples of signal and response time measurements from two monitors, namely the Dell 3007 WFP LCD monitor (Dell Inc., Round Rock, Texas, USA) and the Iiyama HM204DT CRT monitor (Iiyama Corporation, Asakusa-Bashi Taito-Ku, Tokyo, Japan). The Iiyama signal in Fig. 2(a) has been measured by a Tektronix TDS410A oscilloscope, the Dell signal in Fig. 2(b) by an optical transient recorder OTR–3 (Display Metrology & Systems GmbH & Co. KG, Karlsruhe, Germany, http://display-messtechnik.de/typo3/fileadmin/template/main/docs/OTR3-6.pdf).

In order to determine response times shown in Fig. 3, the LCD backlight modulation has been removed according to the division method with dynamical filtering [13]. The bar plots in Fig. 3 show response time averages over five independent measurements for each transition.
